# Patterns and Prevalence of Self-Medication in Saudi Arabia: Insights From a Nationwide Survey

**DOI:** 10.7759/cureus.51281

**Published:** 2023-12-29

**Authors:** Anas Alhur, Afrha Alhur, Amirah Alfayiz, Abdullah Alotaibi, Bushra Hansh, Nada Ghasib, Fahad Alharbi, Nouf Albalawi, Aishah Aljohani, Aseel Almaghthawi, Ahmed Sahlool, Sultan AlThobaiti, Walaa Hakami, Ayed Alghamdi, Zakaria Asiri

**Affiliations:** 1 Health Informatics, University of Hail College of Public Health and Health Informatics, Hail, SAU; 2 Department of Clinical Nutrition, University of Hail, Hail, SAU; 3 Department of Dentistry, Ministry of Health, Riyadh, SAU; 4 Department of General Medicine, Sulaiman Alrajhi University, Riyadh, SAU; 5 Department of Pharmacy, College of Pharmacy, King Khalid University, Asir, SAU; 6 Department of Pharmacy, College of Pharmacy, Jazan University, Jazan, SAU; 7 Optometrist, Eyewa, Riyadh, SAU; 8 Department of Pharmacy, Al-Rayan Medical College, Madinah, SAU; 9 Laboratory Department, Women's, Obstetrics and Children's Hospital, Al-Jawf, SAU; 10 Department of Pharmacy, Al-Rayan Medical College, Riyadh, SAU; 11 Laboratory Department, Madinah Regional Blood Bank, Ministry of Health, Madinah, SAU; 12 Department of Pharmacy, King Abdulaziz Specialist Hospital, Taif, SAU; 13 Department of Public Health, Ministry of Health, Riyadh, SAU; 14 Department of Health Administration and Hospital Management, College of Health Administration and Hospital Management, King Abdulaziz University, Jeddah, SAU

**Keywords:** health education, adverse drug reactions, drug use patterns, public health, self-medication

## Abstract

Self-medication, the unsupervised use of drugs, is a common global behavior with potential adverse health outcomes. This study explores the prevalence and patterns of self-medication in Saudi Arabia, focusing on factors such as drug availability, economic constraints, and public trust in healthcare systems. Particular emphasis is placed on self-medication with antibiotics and prescription drugs due to their significant public health risks. Our comprehensive, quantitative, cross-sectional study surveyed 1,671 individuals across Saudi Arabia's diverse regions. We found that 75.5% of respondents engaged in self-medication, primarily on an occasional basis. While 59.8% of participants perceived self-medication as safe, 17.5% reported experiencing adverse effects. Respondents strongly advocated for increased regulatory measures (87.7%) and a pressing need for enhanced public education (92.6%) to address the associated risks. The study highlights the widespread practice of self-medication in Saudi Arabia, influenced by various factors, and underscores the need for targeted health policies and educational campaigns to mitigate these risks.

## Introduction

Self-medication, as defined by the World Health Organization, involves the use of medicines to treat self-recognized disorders or symptoms without the guidance of a qualified health professional. This practice also includes the intermittent or continued use of medication previously prescribed for chronic or recurring diseases [1]. Across various demographics, self-medication is common, particularly among young people, parents of pediatric patients, and individuals in low- and middle-income countries. Its complexity and potential dangers lie in the risks of adverse drug reactions, antibiotic resistance, and other negative health outcomes [2].

Several factors, including drug availability, economic constraints, cultural practices, previous prescriptions, and media influences, impact the propensity for self-medication. In regions where healthcare services are limited or there is general mistrust in the healthcare system, self-medication often involves antibiotics or prescription medications, heightening the risk of drug resistance and adverse reactions [3].

A high prevalence of self-medication has been observed in India's urban populations, attributed to easy access to pharmacies and lax regulatory frameworks [4]. Among university students in Pakistan, self-medication is driven by factors such as time-saving and prior experiences with similar symptoms [5]. In Brazil, socioeconomic factors, particularly among lower-income groups, lead to self-medication due to the prohibitive costs of healthcare [6]. Rural areas in Argentina also show a tendency towards self-medication, influenced significantly by economic constraints [7].

In Europe, cultural norms and over-the-counter medication availability play a vital role in self-medication practices. In the United Kingdom, self-care and self-medication are common, driven by a desire for independent health management [8]. In Germany, there is a prevalence of self-medication among adults, primarily using non-prescription medications for minor health issues [9]. In the United States, there is an increasing trend in self-medication, especially for managing chronic conditions, with many turning to over-the-counter supplements and herbal remedies [10].

These global trends in self-medication practices underscore the need for region-specific public health strategies that consider the unique cultural, economic, and regulatory factors influencing these behaviors. Addressing the challenges posed by self-medication requires healthcare providers to engage in patient education, focusing on the risks and benefits of self-medication and the importance of professional medical advice. Improving access to healthcare services and ensuring the availability of safe and effective drugs are essential. Policymakers and healthcare professionals must collaborate to develop strategies that balance accessible medication with the risks associated with unsupervised drug use.

This study aims to examine the prevalence and patterns of self-medication in the general population of Saudi Arabia, contributing to the growing body of research on this subject and providing insights into public health interventions and policy development.

## Materials and methods

Study design

This study used a cross-sectional design. The study was conducted from August 23, 2023, to October 18, 2023.

Sampling technique and population

The study population comprised individuals from various demographics across Saudi Arabia.

Stratification Criteria

Demographic factors: Stratified random sampling was based on age, gender, and geographic region. The strata were formed to capture diverse self-medication behaviors influenced by these factors. The proportion of each stratum in the sample was determined based on the population distribution in these categories.

Sample size justification: The sample size of 1,671 participants was calculated to achieve a 95% confidence level with a predetermined margin of error. The expected prevalence of self-medication, based on preliminary studies, was used as the basis for this calculation.

Data collection

Questionnaire Development

The questionnaire was developed collaboratively by a team of academic experts specializing in public health, pharmacy, and epidemiology. Drawing upon a comprehensive review of existing literature on self-medication practices, the team crafted a set of questions that accurately capture the nuances of self-medication behaviors. The final questionnaire encompassed sections on demographic information, patterns of self-medication, reasons for self-medicating, and perceptions of risk. The questions were meticulously designed to be clear, concise, and relevant, employing a mix of multiple-choice and Likert-scale formats to facilitate ease of understanding and response accuracy among participants.

Validity and Reliability

The questionnaire's validity and reliability were ensured through reviews by public health and pharmacy experts.

Pilot testing

A pilot test with a small, diverse subset of the target population was conducted. Feedback was obtained on the clarity, length, and comprehensiveness of the questionnaire, leading to necessary adjustments.

Ethical considerations

Approval Process

The study was reviewed and approved by the Ethical Approval Committee from the Research Department at Hail Health Cluster (Approval No. H-2023-494). This process ensured adherence to ethical standards in research involving human subjects.

Informed Consent

Participants were provided with detailed information about the study's purpose and procedures, and consent was obtained before participation. The consent process emphasized voluntary participation and the confidentiality of responses.

Data Privacy and Security

Data was anonymized and stored securely, with access limited to authorized personnel only.

## Results

Our demographic analysis of the survey participants provided insightful data regarding their backgrounds and characteristics. Of the 1,671 respondents approached, a significant proportion, 1,626 individuals (97.3%), participated in the survey. Only a small number, 14 (0.8%) individuals, declined participation. The gender distribution among participants was predominantly female, with 1,008 female (60.3%) participants and 652 male (39.0%) participants (see Table 1 for detailed demographic distribution). In terms of educational background, the majority of survey participants held a bachelor's degree, with 1,208 (72.3%) individuals falling into this category. This was followed by high school graduates, who accounted for 224 (13.4%) individuals. Participants with education below high school level were the least, consisting of 56 (3.4%) individuals. The geographic distribution of the participants showed a notable concentration in the Western Region, with 694 (41.5%)individuals residing there, and the least representation in the Eastern Province and Central Region (Table 1).

**Table 1:** Participants' demographic distribution

Category	Subcategory	Frequency	Percent
Participation	Yes	1,661	97.30%
	No	14	0.80%
	Total	1,674	98.1%
Gender	Male	652	39.00%
	Female	1,008	60.30%
	Total	1,660	99.3%
Age	18–24	194	11.60%
	25–34	556	33.30%
	35–44	558	33.40%
	45–54	274	16.40%
	More than 54	84	5.00%
	Total	1,660	100.00%
Educational level	Below high school	56	3.40%
	High school graduate	224	13.40%
	Bachelor's	1,208	72.30%
	Master's degree or higher	178	10.70%
	Total	1,660	100.00%
Location	Northern area	374	22.40%
	Southern area	508	30.40%
	Western Region	694	41.50%
	Eastern Province	26	1.60%
	Central Region	62	3.70%
	Total	1,660	100.00%

Our study revealed that a substantial majority of the respondents, 1,262 (75.5%) individuals, reported engaging in self-medication. The mean value for self-medication was 1.24, with a standard deviation (SD) of 0.43, indicating a relatively high consistency among responses. Conversely, 404 (24.2%) participants indicated that they had never self-medicated.

Regarding the frequency of self-medication, the majority of participants, 702 (43%) individuals, reported self-medicating occasionally. This was followed by 320 (19.2%) participants who reported frequent self-medication. The details of these frequencies, along with the mean and SD, are presented in Table 2.

**Table 2:** An overview of self-medication practices

Question	Response	Frequency	Percent	Mean + SD
Have you ever self-medicated?	Yes	1,262	75.5	1.25 ± 0.43
	No	404	24.2	--
	Total	1,671	100	--
How often do you engage in self-medication?	Rarely	242	14.5	--
	Occasionally	702	42	2.35 ± 0.92
	Frequently	320	19.2	--
	Never	216	12.9	--
	Total	1,671	100	--

The perception of self-medication safety varied among participants. A majority, 1,000 (59.8%) individuals, believed self-medication to be a safe practice, with a mean score of 1.39 and an SD of 0.49. However, 292 respondents (17.5%) reported experiencing adverse effects from self-medication, with a mean score of 1.82 and an SD of 0.38.

A significant majority, 1,466 (87.7%) participants, expressed the need for more regulations and guidelines for self-medication, with a mean score of 1.11 and an SD of 0.31. Awareness of the risks and dangers associated with self-medication was also high, with 1,230 (73.6%) participants acknowledging these risks (Table 3).

**Table 3:** Factors influencing self-medication

Question	Response	Frequency	Percent	Mean + SD
Do you believe self-medication is a safe practice?	Yes	1,000	59.8	1.39 ± 0.49
	No	638	38.2	--
	Total	1,671	98	--
Have you ever experienced any adverse effects from self-medication?	Yes	292	17.5	1.82 ± 0.39
	No	1,330	79.6	--
	Total	1,622	97.1	--
Do you think there should be more regulations and guidelines for self-medication?	Yes	1,466	87.7	1.11 ± 0.31
	No	172	10.3	--
	Total	1,671	100	--

The survey also revealed that approximately half of the participants, 834 (49.9%) individuals, had sought professional advice after self-medicating, with a mean score of 1.26 and an SD of 0.44. Furthermore, a substantial majority, 1,548 (92.6%) participants, expressed the opinion that there should be more public education on the risks and proper practices of self-medication, as detailed in Table 4.

**Table 4:** Awareness and education on self-medication

Question	Response	Frequency	Percent	Mean + SD
Are you aware of the potential risks and dangers of self-medication?	Yes	1,230	73.6	1.11 ± 0.31
	No	426	25.5	--
	Total	1,671	100	--
Have you ever sought professional advice after self-medicating?	Yes	834	49.9	1.26 ± 0.44
	No	790	47.3	--
	Total	1,671	100	--
Do you think there should be more public education on the risks and proper practices of self-medication?	Yes	1,548	92.6	1.49 ± 0.49
	No	96	5.7	--
	Total	1,671	100	--

These findings underline the prevalent practice of self-medication in Saudi Arabia, with significant numbers indicating both a general perception of its safety and the experience of adverse effects, highlighting the need for enhanced public education and regulation.

**Figure 1:**
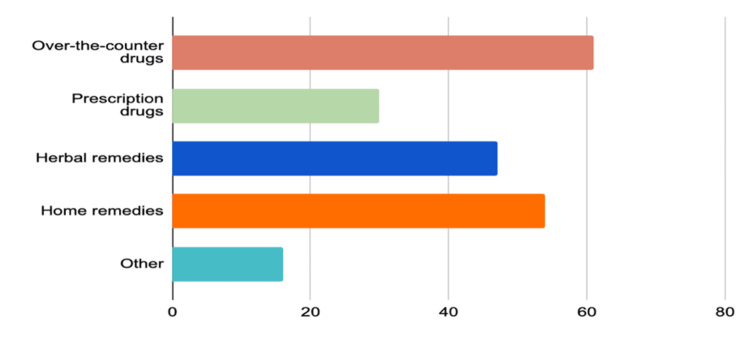
Type of substances used

Figure 1 provides a visual representation of the usage percentages for various remedy types. Over-the-counter drugs lead with a usage rate of 61%, indicating they are the most commonly used category among the options listed. Prescription drugs are used 30% of the time. Herbal remedies also have a considerable usage rate of 47%, suggesting a preference for natural treatment options among a substantial portion of the population. Home remedies, with a 54% usage rate, indicate a strong inclination towards self-managed care. Lastly, the “Other” category accounts for 16% usage, which could include a variety of less common or unclassified types of remedies.

**Figure 2:**
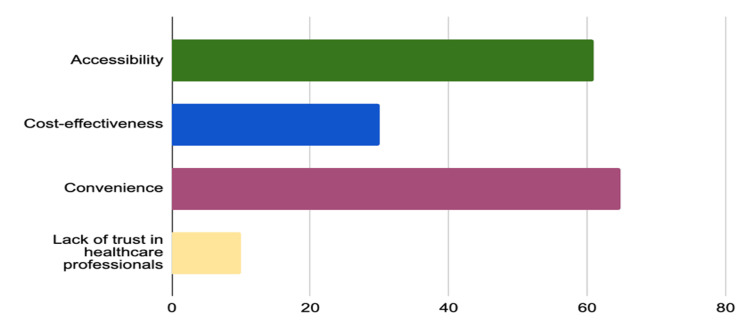
Factors influencing self-medication

Figure 2 shows the various factors that influence self-medication. Accessibility is the leading factor at 61%, suggesting that the ease of obtaining medications plays a significant role. Convenience is also a major influencer, with 64.80%, indicating that the ability to self-medicate at one's own discretion is highly valued. Cost-effectiveness, at 30%, shows that economic considerations are important but not the primary driver. Meanwhile, a lack of trust in healthcare professionals is a factor for 10% of individuals, pointing to some dissatisfaction with formal medical advice. Other unspecified factors also contribute to the decision to self-medicate.

**Figure 3:**
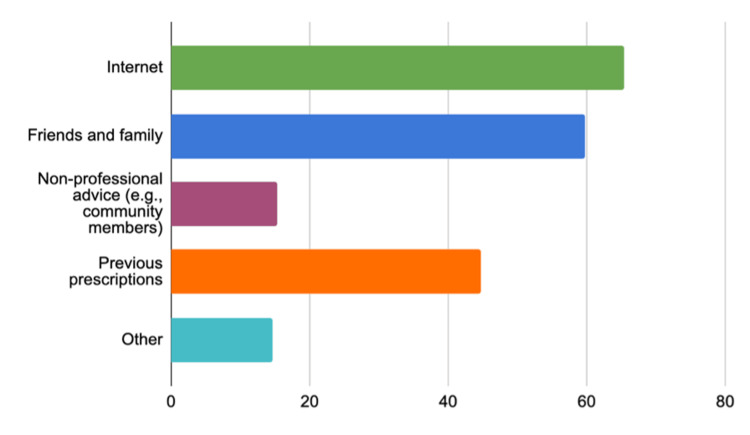
Sources of information

Figure 3 shows the sources from which individuals commonly gather information for self-medication. The Internet is the most used source at 65.40%, indicating a strong preference for online research. Friends and family are also a significant source of information, cited by 59.70% of individuals, reflecting reliance on personal networks. Previous prescriptions inform 44.70% of self-medication decisions, suggesting that past medical advice continues to influence current choices. Non-professional advice, such as that from community members, plays a smaller role at 15.30%. Lastly, other unspecified sources account for 14.60% of the information used for self-medicating.

A full list of the survey questions referenced in the study can be found in Appendix A and Appendix B, providing a context for the responses analyzed.

## Discussion

This study's analysis of self-medication within Saudi Arabia's general population reveals extensive practices reflective of international trends, aligning with findings from diverse geographies [8,9]. A significant 75.5% of respondents reported self-medicating, a figure that resonates with the broader, documented prevalence of self-medication behaviors [11,12]. Particularly revealing is the high incidence of self-medication among students, where 92.3% across various disciplines reported self-treating within the study's timeframe [13].

The public's perception of the safety of self-medication is divided, with 59.8% viewing it as safe, contrasting sharply with the 38.2% who perceived it as unsafe. This dichotomy underscores the imperative for comprehensive public health education to address misconceptions and encourage responsible self-medication practices [14]. The widespread practice of self-medication is recognized globally as a potential pathway to adverse outcomes, including the risk of developing antibiotic resistance, which further emphasizes the need for education on the responsible use of medications [15].

Adverse effects from self-medication were reported by 17.5% of our study's participants, echoing previous research highlighting the risks associated with unsupervised medication use [16]. This substantiates the critical need for informed decision-making, supported by the 87.7% of respondents who voiced the necessity for more stringent regulations and guidelines [17].

It is encouraging to note that 73.6% of participants were cognizant of the risks associated with self-medication, indicating a baseline of awareness conducive to fostering safer self-medication practices. Moreover, nearly half of the respondents sought professional advice post-self-medication, showcasing a commendable level of responsibility in personal healthcare management. The overwhelming majority, 92.6%, calls for more public education on the risks and proper practices of self-medication, a sentiment that echoes the necessity for targeted educational campaigns similar to those implemented in other countries [18,19].

This study provides valuable insights into the self-medication practices within Saudi Arabia, aligning with international trends and providing a comprehensive understanding of this phenomenon in a specific cultural and healthcare context. One of the key strengths of this study is its large sample size and the use of stratified random sampling, which ensures diverse demographic representation. Additionally, the study's design allows for a detailed exploration of self-medication patterns, perceptions of safety, and associated behaviors among the general population.

However, there are limitations to this study that should be acknowledged. The reliance on self-reporting can introduce biases such as recall bias and social desirability bias, which may affect the accuracy of the data. The study's findings are specific to Saudi Arabia and may not be generalizable to other cultural or healthcare settings, suggesting a need for caution when extrapolating these results. Furthermore, the cross-sectional nature of the study limits our ability to establish causal relationships between observed factors and self-medication practices.

Despite these limitations, the study's findings are significant and offer a foundation for future research. They underline the need for an integrated approach that includes stricter regulations, comprehensive public education, and awareness campaigns to mitigate the risks associated with self-medication. Such strategies are crucial for enhancing informed health decisions and improving public health outcomes.

## Conclusions

This study illuminates the prevalence of self-medication in Saudi Arabia, revealing that 75.5% of the population has engaged in this practice, underscoring its ubiquity across varied demographics. Despite 59.8% of participants believing in the safety of self-medicating, the 17.5% who experienced adverse effects highlight a dangerous gap in public awareness about the potential risks.

The data reflects an unequivocal public call for stricter regulations and enhanced educational outreach on self-medication. A considerable 87.7% of participants advocate for more robust regulatory measures, and a higher percentage, 92.6%, recognize the need for increased public education to safeguard health. These findings point to an urgent necessity for policy reforms and public health initiatives that focus on mitigating the risks of self-medication through improved patient education and access to healthcare services.

## Figures and Tables

**Figure 4:**
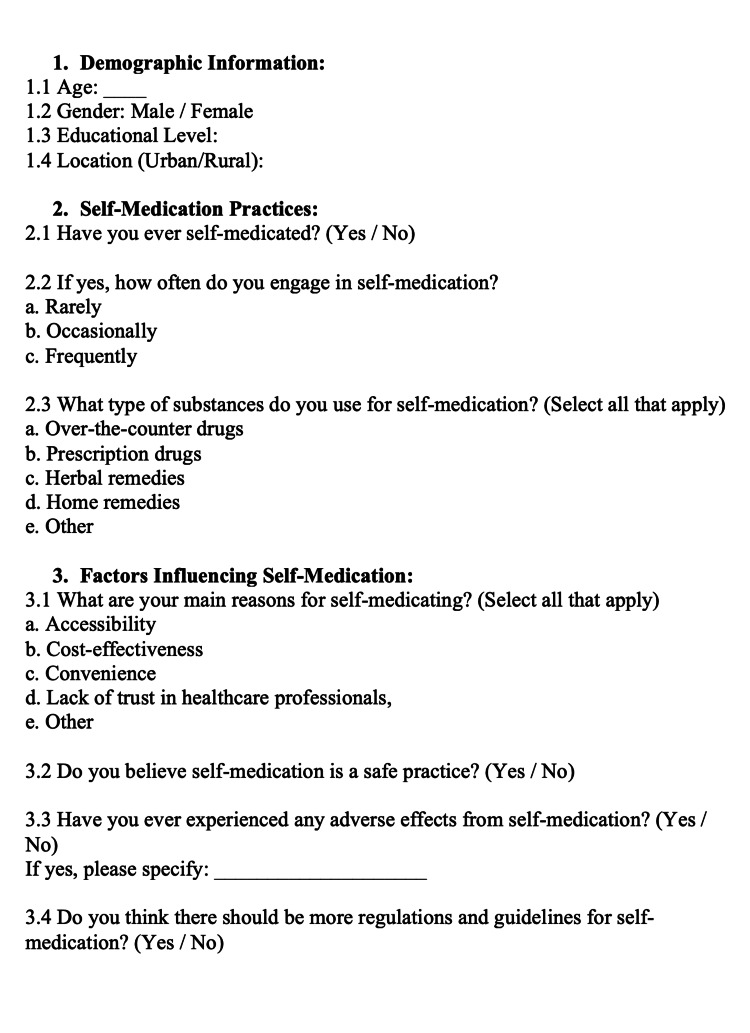
Self-medication survey questionnaire

**Figure 5:**
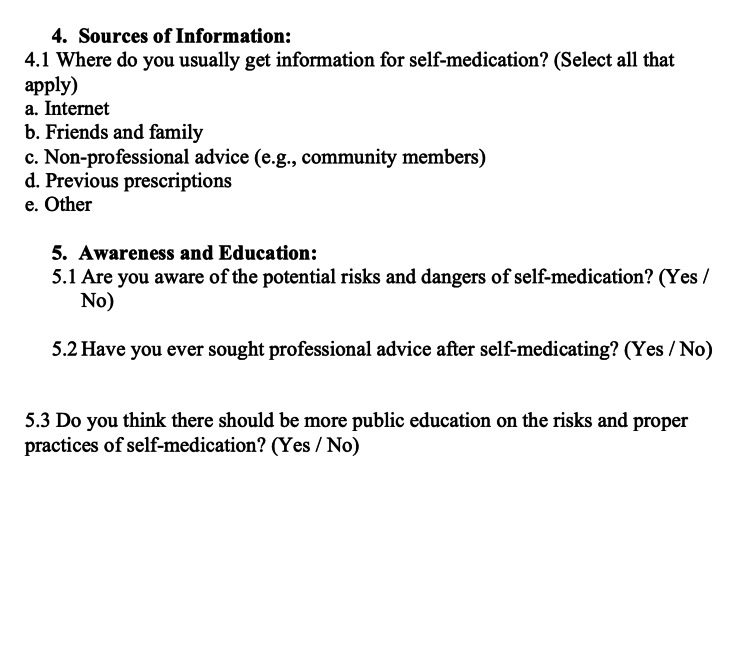
Self-medication survey questionnaire

## Appendices

Appendix A 

Appendix B 
